# Recurrent seizure-like events are associated with coupled astroglial synchronization

**DOI:** 10.3389/fncel.2015.00215

**Published:** 2015-06-18

**Authors:** Orsolya Kékesi, Enikö Ioja, Zsolt Szabó, Julianna Kardos, László Héja

**Affiliations:** Research Centre for Natural Sciences, Hungarian Academy of Sciences, Institute of Organic Chemistry, Functional Pharmacology GroupBudapest, Hungary

**Keywords:** astrocytes, synchronization, neuro-glia coupling, epilepsy, Ca^2+^ imaging, gap junctions

## Abstract

Increasing evidence suggest that astrocytes significantly modulate neuronal function at the level of the tripartite synapse both in physiological and pathophysiological conditions. The global control of the astrocytic syncytium over neuronal networks, however, is still less recognized. Here we examined astrocytic signaling during epileptiform activity which is generally attributed to large-scale neuronal synchronization. We show that seizure-like events in the low-[Mg^2+^] *in vitro* epilepsy model initiate massive, long-range astrocytic synchronization which is spatiotemporally coupled to the synchronized neuronal activity reaching its maximum at the electrographic tonic/clonic transition. Cross-correlation analysis of neuronal and astrocytic Ca^2+^ signaling demonstrates that high degree of synchronization arises not only among astrocytes, but also between neuronal and astrocyte populations, manifesting in astrocytic seizure-like events. We further show that astrocytic gap junction proteins contribute to astrocytic synchronization since their inhibition by carbenoxolone (CBX) or Cx43 antibody increased the interictal interval and in 41% of slices completely prevented recurrent seizure-like activity. In addition, CBX also induced unsynchronized Ca^2+^ transients associated with decreasing incidence of epileptiform discharges afterwards. We propose therefore that local, unsynchronized astrocytic Ca^2+^ transients inhibit, while long-range, synchronized Ca^2+^ signaling contributes to the propagation of recurrent seizure-like events.

## Introduction

Astrocytes are increasingly recognized as important players in the modulation of physiological neuronal function and various pathophysiological conditions and diseases (Verkhratsky et al., 2013, 2014a,b,c, 2015). In addition to modulation by the supplement or lack of metabolic support, they also directly control neuronal activity. In particular, growing evidence demonstrate the involvement of astroglia in epileptiform activity by various local and long-range mechanisms (Tashiro et al., 2002; Fellin et al., 2006; Wetherington et al., 2008; Gómez-Gonzalo et al., 2010). Local astroglial modulation of synaptic activity includes the classical glutamate clearance, but several other processes as well: release of transmitters (Bezzi et al., 1998; Guthrie et al., 1999; Zhang et al., 2003; Lee et al., 2010), exchange of excitatory glutamate (Glu) to γ-amino-butyric acid (GABA; Héja et al., 2009, 2012; Dvorzhak et al., 2013; Unichenko et al., 2013) and astrocytic osmoregulation (Pál et al., 2013). In addition, astrocytes are heavily interconnected through gap junctions and the astrocyte syncytium is able to control neuronal function on longer spatial scales by buffering K^+^, spreading synchronization (Amzica and Massimini, 2002; Timofeev et al., 2002) or distributing metabolic energy supply (Kovács et al., 2012). Importantly, long-range astrocytic activation may also be coupled to the regulation of neurovascular function (Gómez-Gonzalo et al., 2011). Disruption of this long-range astrocytic coupling was found to be ambiguous: gap junction blockers were showed to suppress seizure-like activities both *in vitro* (Jahromi et al., 2002; Nyikos et al., 2003; Samoilova et al., 2003) and *in vivo* (Elisevich et al., 1998), but permanent knock out of the gap junctional proteins Cx43 and Cx30 initiated spontaneous epileptiform events (Wallraff et al., 2006).

Astrocytes respond to neuronal activity by Ca^2+^ transients that can be used as a readout of astrocyte activation. These calcium oscillations are transmitted to neighboring astrocytes by either gap junctional IP_3_ transmission or connexin hemichannel-mediated ATP release (Hirase et al., 2004; Kuga et al., 2011; Molnár et al., 2011a,b). The resulting calcium waves propagate *in vitro* (Parri et al., 2001; Nett et al., 2002) and *in vivo* (Hirase et al., 2004; Kuga et al., 2011) during both epileptiform and interictal activity (Kovács et al., 2000; Nyikos et al., 2003; Carmignoto and Haydon, 2012). There is, however, an ongoing debate whether glial Ca^2+^ signaling precedes the seizure-like events (Carmignoto and Haydon, 2012), appears during interictal discharges (Tian et al., 2005) or rather follows seizure onset (Gómez-Gonzalo et al., 2010), addressing different stages of the epileptiform activity. Importantly, despite the relatively widely studied role of astrocytes in ictogenesis, very little is known about astrocytic contribution to the termination of seizures.

Synchronization, implied in both seizure genesis and termination is a hallmark of the epileptic condition that involves various synaptic and non-synaptic signaling mechanisms (Kramer et al., 2012; Jiruska et al., 2013). Among these, astrocytes may play a prominent role in the global regulation of neuronal networks (Nedergaard and Verkhratsky, 2012) since they are capable of: (1) detecting neuronal activity; (2) responding to this activity by local Ca^2+^ transients; (3) propagating the local changes to extended spatial scales through Ca^2+^ waves; and (4) stimulating neuronal activity at multiple locations by gliotransmitter release or regulation of ion homeostasis.

Here we addressed the contribution of astrocytes to recurrent epileptiform bursting in the low-[Mg^2+^] *in vitro* epilepsy model by determining the spatiotemporal correlation between neuronal and astroglial activity as well as by measuring the effects of local neuron-astroglia interaction and long-range astrocytic coupling on astrocyte Ca^2+^ signaling. We provide evidence that astrocytes significantly contribute to the long-range propagation of synchronized neuronal and epileptiform activity.

## Materials and Methods

Animals were kept and used in accordance with the European Council Directive of 24 November 1986 (86/609/EEC) and the Hungarian Animal Act, 1998 and associate guidelines. All efforts were made to minimize animal suffering and the number of animals used. All drugs were obtained from Sigma unless stated otherwise.

### Buffers

Oxygenated (95% O_2_, 5% CO_2_) artificial cerebrospinal fluid (ACSF) contained in mM: 129 NaCl; 1.23 NaH_2_PO_4_; 10 glucose; 1.6 CaCl_2_.H_2_O; 3 KCl; 21 NaHCO_3_; 1.8 mM MgSO_4_. To induce epilepsy MgSO_4_ was eliminated and 2 mM KCl was added (low-[Mg^2+^] ACSF). Stock solutions of cell permeant Fluo-4 AM (2.5 mM, Life Technologies) and Oregon Green BAPTA-1 AM (OGB-1, 800 μM, Life Technologies) were diluted in 20% Pluronic F-127 (Life Technologies). Final DMSO concentration was 0.16% (Fluo-4) or 0.2% (OGB-1), which did not significantly altered epileptiform activity. Astrocytic γ-aminobutyric acid (GABA) transporter inhibitor SNAP-5114 (Tocris) stock solution (100 mM) was diluted in DMSO (final DMSO concentration in these experiments: 0.1%). Stock solution (50 mM, Sigma) of carbenoxolone hemisuccinate (CBX) was diluted in distilled water. Stock solution of astroglia-specific marker dye sulforhodamine101 (SR101, 10 mM, Invitrogen) was diluted in distilled water.

### Slice Preparation

Transverse, 400 μm thick hippocampal-entorhinal cortex slices from P12–14 male Wistar rats (Toxicoop, Hungary) were prepared with Leica vibratome (Leica-VT 1000S). After decapitation, brains were quickly removed and put into an oxygenated (95% O_2_, 5% CO_2_), ice cold solution of modified artificial cerebrospinal fluid: 75 mM sucrose; 87 mM NaCl; 2.5 mM KCl; 1.25 mM NaH_2_PO_4_; 7 mM MgSO_4_; 0.5 mM CaCl_2_; 25 mM NaHCO_3_; 25 mM glucose. Slices were transferred to an interface type chamber and incubated in continuously oxygenated ACSF at 37°C for 1 h.

### *In Vitro* Electrophysiology

Spontaneously recurrent SLEs were evoked by changing the perfusion solution to a nominally Mg^2+^ free (based on the Mg^2+^ contamination of the Ca^2+^ salts, we estimate the Mg^2+^ concentration of this buffer to be ~1 μM) ACSF solution with [K^+^] elevated to 5 mM (low-[Mg^2+^] ACSF). For field potential (FP) recordings glass microelectrodes (3–6 MΩ) were filled with low-[Mg^2+^] ACSF solution and were inserted in the CA3 stratum pyramidale. Signals were recorded with Multiclamp 700A amplifiers (Axon Instruments, Foster City, CA, USA) and digitized at 10 kHz (Digidata1320A, Axon Instruments). Slices were discarded if SLE did not appear in 20 min starting from the application of low-[Mg^2+^] ACSF. Electrographic tonic-to-clonic transitions were identified by the first appearance of secondary discharges (Lasztóczi et al., 2009). Recordings were analyzed after high-pass filtering at 1 Hz.

### Ca^2+^ Imaging

Fluorescence recordings of Fluo-4 loaded hippocampal slices were made with an upright microscope (Olympus BX61WI) equipped with a FluoView300 confocal laser-scanning system (Olympus, Tokyo, Japan) using a 40× water immersion objective (N.A. 0.80). OGB-1 imaging was performed using a two-photon microscope (Femtonics, Hungary) equipped with a 10× water immersion objective (N.A. 0.30). For SR101 imaging, slices were incubated right after slicing by changing the normal ACSF solution in the interface type chamber to an ACSF containing 1 μM SR101 for 20 min at 37°C (Kafitz et al., 2008; Héja et al., 2012). For Fluo-4 and OGB-1 imaging, slices were incubated with 5 μM Fluo-4 AM or 10 μM OGB-1 AM in ACSF at 37°C for 1 h in the dark under continuously oxygenated atmosphere after the initial 1 h incubation in the interface type chamber (Lasztóczi et al., 2006). Slices were transferred into a submerge-type recording chamber mounted on the stage of the microscope and were superfused with oxygenated ACSF (3 ml/min, ~30°C). Images acquired from CA3 stratum radiatum were taken every 5 s for Fluo-4 measurements and every 40 ms (92 cells) to 140 ms (256 cells) for OGB-1 measurements. Occasionally, focus was recalibrated during the experiments due to objective shifting, during which period, no images were taken. Fluo-4 was excited at 488 nm and emitted fluorescence was monitored using a 510–530 nm bandpass filter. SR101 was excited at 543 nm and emitted fluorescence was monitored at 570–600 nm. In OGB-1 measurements both OGB-1 and SR101 were excited at 900 nm with a Mai Tai femtosecond laser source (Spectra Physics, USA) and emitted fluorescence was monitored at 475–575 nm (OGB-1) and 600–700 nm (SR101).

### Drug Application

Drugs were always applied following the first SLE. CBX and SNAP-5114 were continuously present for the next two SLEs after which the experiments were terminated. Cx43 antibody (Abcam, #ab11370) was applied according to a different protocol: to avoid excessive consumption of the antibody in a continuously superfused medium, perfusion of ACSF was stopped after the first SLE and Cx43 antibody was added to the chamber in a relatively high concentration (7.5 μg/ml, 1:100 dilution). After 10 min, the perfusion was restarted and the antibody was washed out. In control measurements, the same protocol was applied without adding Cx43 antibody after perfusion was stopped.

Parameters calculated for drug applications were compared to the same order (second and third) control SLEs.

### Data Processing

Cells on Fluo-4 and OGB fluorescence images were identified by semi-automatic Matlab scripts under manual supervision. The identified regions of interests (ROIs) contained the soma of the cells which was slightly extended by 2–3 pixels in all directions to reduce the noise in fluorescence intensity. The ROIs were visually validated and the cell type (neuron or astrocyte) was determined based on the absence or presence of SR101 labeling in the same ROI. Average intensity in the identified ROIs (containing the soma, but not the processes of the cells) were then calculated followed by subtraction of and dividing by the average intensity of ROIs in the first 10 s. The resulting ΔF/F_0_ traces were detrended and fluorescence peaks were identified using 5*SD as detection threshold (Molnár et al., 2011a). Detection threshold was selected after manually revising calculated ΔF/F_0_ traces from 29 slices. The criteria for threshold selection was to eliminate false peak detection during the automated peak finding process and therefore to avoid artificial introduction of synchronous events.

Neuronal and astrocytic synchronization was calculated by two different methods. Average synchronization during a specific condition, like preictal phase or SLE was determined by calculating the cross-correlation coefficients for the ΔF/F_0_ traces of each pair of cells. To determine the temporal evolution of synchronization, another method was applied: the number of cells having a fluorescence peak in a 10 s (Fluo-4 measurements) or 1 s (OGB-1 measurements) wide moving window was divided by the number of validated cells in the field of view giving the ratio of synchronization in percentage.

Unless stated otherwise data are expressed as means ± S.E.M. and were analyzed using one-way analysis of variances with Bonferroni *post hoc* tests (OriginPro 8.0). A value of *P* < 0.05 was considered significant.

## Results

### Astrocytes Synchronize After the Onset of Seizure-Like Events

**Figure 1:**
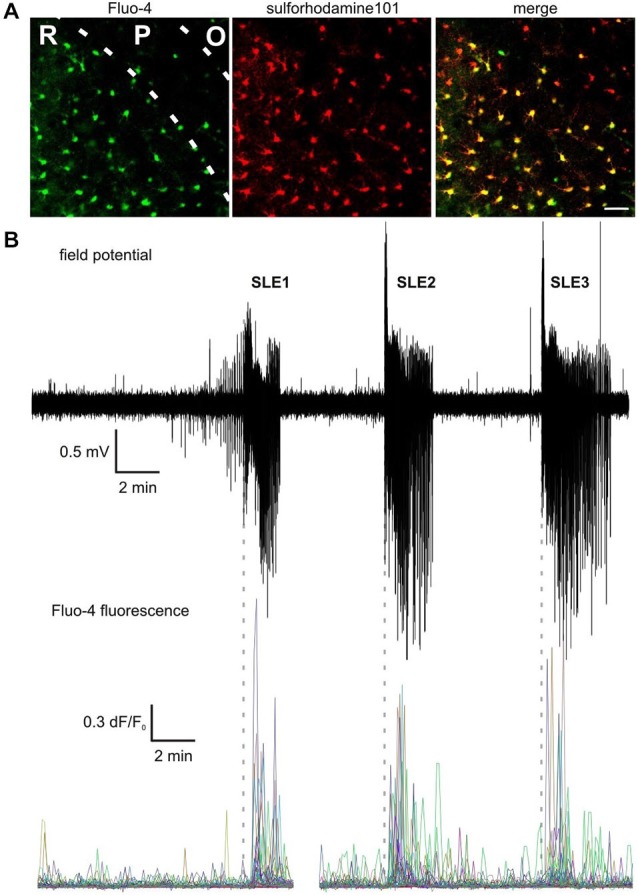
**Astrocytes show increased Ca^2+^ signaling during seizure-like events.** (**A**) Colocalization of Fluo-4 (left) and sulforhodamine-101 (middle) labeling. R: str. radiatum, P: str. pyramidale, O: str. oriens. Scale bar: 20 μm. (**B**) (Top) Extracellular field potential recording of seizure-like events (SLEs) in the CA3 region in a hippocampal slice. (Bottom) Ca^2+^ transients measured by Fluo-4 fluorescence in CA3 astrocytes. Vertical dashed lines indicate the start of SLEs.

**Figure 2:**
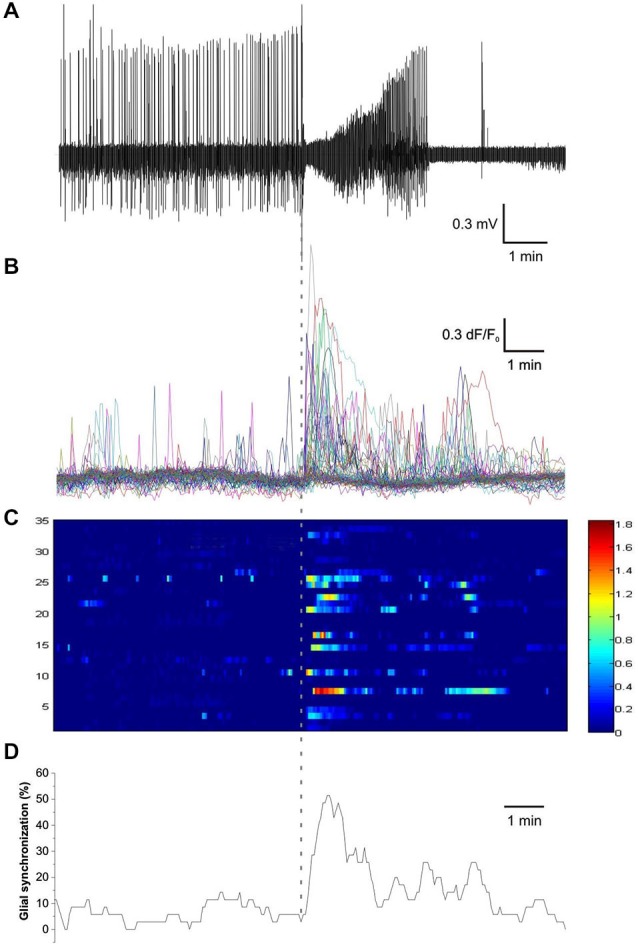
**Astrocytes synchronize their activity after the initiation of seizure-like events. (A)** Extracellular field potential recording of a seizure-like event (SLE) in the CA3 region in a hippocampal slice. **(B)** Ca^2+^ transients measured by Fluo-4 fluorescence in CA3 astrocytes during SLE. **(C)** Ca^2+^ transients in individual astrocytes. Each row represents a single astrocyte. **(D)** Percentage of astrocytes showing Ca^2+^ transients within a 10 s window. Vertical dashed line indicates the start of SLE.

We monitored astrocytic activity in the CA3 region of rat acute hippocampal slices during seizure-like events (SLEs) by Fluo-4 fluorescence. Astrocytic loading of Fluo-4 was confirmed by simultaneous labeling with the astrocyte specific (Nimmerjahn et al., 2004; Kafitz et al., 2008) dye sulforhodamine-101 (SR101) and visual inspection for astrocytic morphology (Figure 1A). In average 50 ± 8 cells were loaded in the recording field of view (354 × 354 μm area) in the CA3 *str. radiatum* and *str. pyramidale*, of which 93 ± 4% were found to be colocalized with SR-101. Epileptiform activity was induced by applying nominally Mg^2+^-free ACSF. In the presence of low-[Mg^2+^] ACSF, recurrent SLEs, characterized by SLE onset, tonic-clonic phases, SLE termination and—in some cases—paroxysmal depolarization shifts before the onset of SLEs appeared in juvenile rat hippocampus and monitored by an extracellular FP electrode in the CA3 region (Figure 1B). Under control conditions (normal ACSF), astrocytes exhibited spontaneous Ca^2+^ oscillations in accordance with previous reports (Parri et al., 2001; Nett et al., 2002; Wang et al., 2006), although these Ca^2+^ signals were not correlated to FP activity (Figure 1B). However, shortly after the onset of SLEs, a large portion (51 ± 7%, *n* = 61 slices) of astrocytes showed massive Ca^2+^ transients. In addition to the increased frequency, also demonstrated in the low-[Mg^2+^]/picrotoxin model of epilepsy (Gómez-Gonzalo et al., 2010), we observed substantial enhancements in the amplitude of astrocytic Ca^2+^ transients (Figures 1B, 2B). Average frequency increased from 0.075 ± 0.013 1/min in the preictal phase to 0.163 ± 0.022 1/min during the first SLE (*p* < 0.001). Average peak amplitude of the ΔF/F_0_ traces increased from 0.14 ± 0.02 in the preictal phase to 0.30 ± 0.08 during the first SLE (*p* = 0.04). It is to note that although removal of Mg^2+^ may itself initiate spontaneous Ca^2+^ signaling (Stout and Charles, 2003), we never observed appearance of spontaneous Ca^2+^ waves during the application of low-[Mg^2+^] ACSF in the absence of SLE activity and neither the frequency nor the amplitude of the Ca^2+^ transients changed significantly between the control phase (normal ACSF) and the preictal phase (low-[Mg^2+^] ACSF).

In addition to the increased frequency and amplitude of astrocytic Ca^2+^ events during SLEs, it was also clear that multiple astrocytes showed Ca^2+^ elevations shortly after the SLE onset. To quantify the temporal evolution of this apparent synchronization of astrocytic activity we calculated the number of astrocytes showing fluorescence peaks (ΔF/F_0_ > 5*SD) in a 10 s wide moving time window and divided it by the total number of labeled astrocytes in the field of view giving the ratio of astrocytic synchronization (Figure 2). Glial synchronization reached 50 ± 5%, 45 ± 5%, and 44 ± 6% in the first three consecutive SLEs, respectively (average of maximal synchronizations in *n* = 31 slices), demonstrating that a high ratio of total astrocytes are synchronously activated after SLE onset. Interestingly, the timing of maximal astrocytic synchronization was found to be well correlated to the transition between the tonic and clonic phases of SLEs. The delay between the maximal astrocytic synchronization and the electrographic tonic/clonic transition was −3 ± 4, −4 ± 6 and 3 ± 6 s in the first three SLEs (Figure 5C).

### Correlation of Neuronal and Astrocytic Synchronization

**Figure 3:**
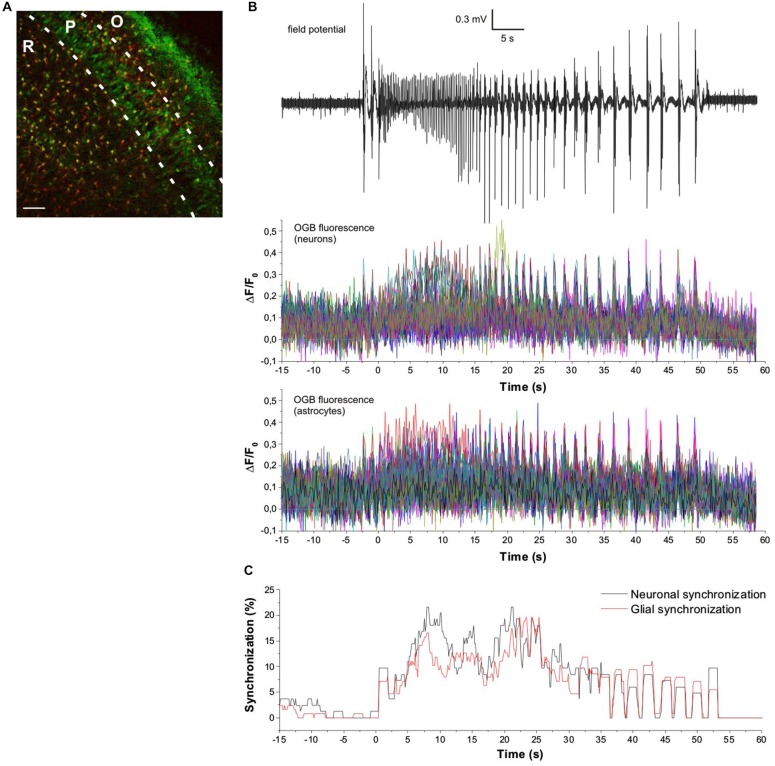
**Astrocytes and neurons are co-synchronized during SLE. (A)** Fluorescent labeling of hippocampal neurons and astrocytes with OGB (green) and simultaneous identification of astrocytes with sulforhodamine-101 (red). R, str. radiatum; P, str. pyramidale; O, str. oriens. Scale bar: 40 μm. **(B)** Neuronal and astrocytic activity monitored by OGB fluorescence during a seizure-like event (SLE). SLE is detected by extracellular field potential recording electrode placed in the CA3 region of the hippocampus (top). Neuronal and astrocytic activity are simultaneously measured from identified neurons (middle) and astrocytes (bottom). Fluorescent changes are shown as dF/F_0_ traces. *t* = 0 corresponds to the onset of SLE. **(C)** Synchronization of neuronal and glial cells during an SLE. Synchronization is expressed as the percentage of neurons or astrocytes having peak fluorescence in a sliding 1 swide window. *t* = 0 corresponds to the onset of SLE.

Neuronal synchronization is a hallmark of SLEs which is manifested in large amplitude transients on the FP recordings. However, in our experiments FP recording was performed at a single location, therefore it does not give information about the spatial domain of the neuronal synchronization. To compare spatiotemporal neuronal and astroglial synchronizations we performed Ca^2+^ imaging simultaneously in both cell types. We used Oregon Green BAPTA (OGB) which, in contrast to the preferential astrocyte labeling of Fluo-4, loads into both neurons and astroglial cells (Ikegaya et al., 2005; Figure 3A). Neurons and astrocytes were separated by SR101 labeling and OGB fluorescence was detected from identified neurons and astrocytes (Figure 3B). On average 210 ± 45 cells were loaded in the recording field of view (600 × 600 μm area) in the CA3 that contained both the *str. radiatum* and *str. pyramidale*. 60 ± 8% of OGB loaded cells were found to be colocalized with SR101, demonstrating that OGB loads both into neurons (pyramidal cells as well as interneurons) and astrocytes (Figure 3A), in contrast to Fluo-4 of which 93% was colocalized with the astrocytic marker SR101. To further investigate the temporal profile of astrocytic synchronization, these experiments were carried out in line scan mode, therefore reducing the sampling interval from 1.2 s to the range 40–140 ms depending on the number of measured cells.

Similarly to the Fluo-4 measurements, both neurons and astrocytes were found to be activated and highly synchronized after the SLE onset (Figure 3B). Due to the increased sampling frequency, significant astrocytic synchronization was also observed corresponding to the preictal and clonic discharges (Figure 3B). The temporal profile of astrocytic and neuronal synchronization was assessed by calculating the ratio of astrocytes or neurons having fluorescence peaks in a certain moving time window. However, contrary to the Fluo-4 measurements, the width of the time window was reduced from 10 s to 1 s owing to the increased sampling frequency. Even on this shorter time scale, astrocytic activity appeared to be synchronized, since up to 20% of all identified astrocytes in the field of view exhibited simultaneous Ca^2+^ signals.

**Figure 4:**
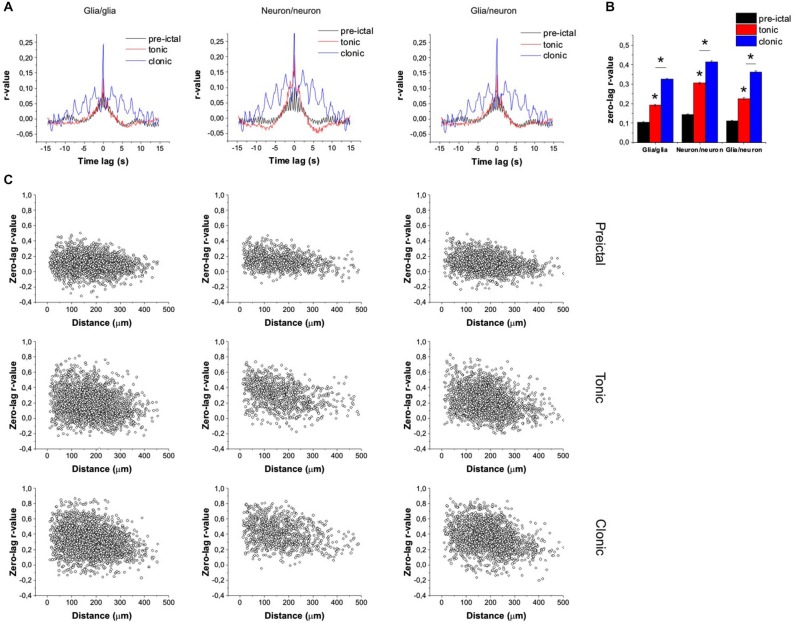
**Co-synchronization of neurons and astrocytes evolves during SLE.** (**A**) Average pair-wise cross-correlograms of neuronal and astroglial dF/F_0_ traces during control conditions and in the tonic and clonic phases of SLE. (**B**) Average zero-lag cross-correlation coefficients during control conditions and in the tonic and clonic phases of SLE. Asterisks denote significant differences. **(C)** Distance dependence of zero-lag cross-correlation coefficients during control conditions and in the tonic and clonic phases of SLE.

To explore the details of neuronal and glial synchronization, we calculated the pair-wise cross-correlograms of neuronal and astrocytic ΔF/F_0_ traces. In the preictal phase (60 s before the SLE onset), only a low-level correlation was observed (Figures 4A,B) among glial cells (average of *N* = 8065 pairs) or neurons (average of *N* = 3445 pairs). Glial vs. neuronal activity was also only modestly correlated (average of *N* = 5270 pairs). However, cross-correlation in all groups was significantly increased during SLE showing high degree of synchronous activity (Figures 4A,B). Correlation between neuronal and astrocytic activity propagation was explored by determining the effect of cell pair distances on the zero-lag cross-correlation coefficients. In the preictal phase the correlation coefficients were uniformly distributed among neighboring and distal neurons or astrocytes and also between the two different cell types, confirming that spontaneous activity show no local and long-range synchronization (Figure 4C, top). During SLE, however, the significant increase in correlation corresponded to a clear distance-dependance, demonstrating that neighboring cells are more synchronized both in the tonic and the clonic phase (Figure 4C, bottom). Importantly, cross-correlation of glial vs. neuronal cells showed the same degree and distance dependance of synchronization as of neuronal vs. neuronal cells, suggesting that astrocytic Ca^2+^ waves might support neuronal long-range synchronization.

### Blockade of Gap Junctions

**Figure 5:**
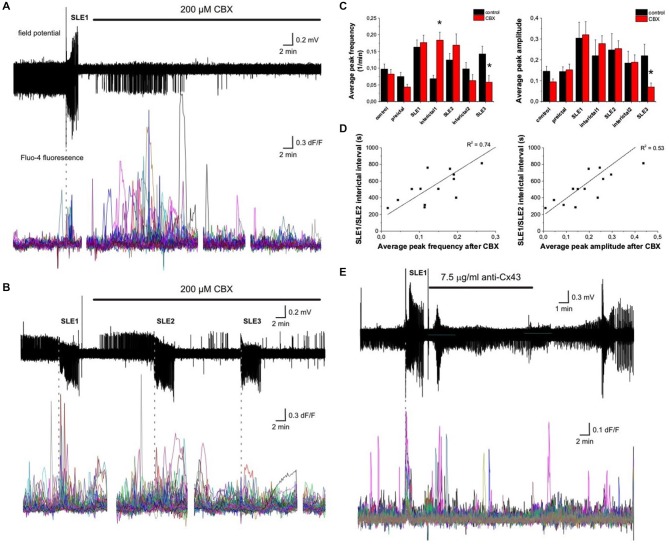
**Anti-convulsant effect of gap junction blockade by gap junction inhibition.** (**A**) (Top) Extracellular field potential recording of a seizure-like event (SLE) in the CA3 region in a hippocampal slice. 200 μM carbenoxolone (CBX) prevents the appearance of additional SLEs. (Bottom) Ca^2+^ transients measured by Fluo-4 fluorescence in CA3 astrocytes. Vertical dashed line indicates the start of SLE. (**B**) (Top) Extracellular field potential recording of SLEs in the CA3 region in a hippocampal slice. 200 μM CBX does not prevent the appearance of additional SLEs, but increases the interictal interval. (Bottom) Ca^2+^ transients measured by Fluo-4 fluorescence in CA3 astrocytes. Vertical dashed lines indicate the start of SLEs. (**C**) Average peak frequency (left) and peak amplitude (right) during the control period, the application of low-[Mg^2+^] ACSF (preictal) and the SLEs and interictal periods. Peak frequency is significantly increased in the first interictal period after the administration of 200 μM CBX. Asterisks denote significant differences between the control conditions and CBX application. (**D**) Relationships between Ca^2+^ peak frequency (left) or amplitude (right) and length of interictal intervals after the first SLE in *N* = 13 slices in which further SLEs appeared during CBX application. In *N* = 7 slices 200 μM CBX completely eliminated SLEs (average peak frequency = 0.23 ± 0.07 1/min; average peak amplitude = 0.32 ± 0.11 in these slices). (**E**) (Top) Extracellular field potential recording of a seizure-like event (SLE) in the CA3 region in a hippocampal slice. 7.5 μg/ml (1:100 dilution) Cx43 antibody prevents the appearance of additional SLEs. (Bottom) Ca^2+^ transients measured by Fluo-4 fluorescence in CA3 astrocytes. Vertical dashed line indicates the start of SLE.

The appearance of synchronous astrocytic activity and its contribution to SLEs offers the possibility to intervene into seizure propagation by blocking the long-range coupling between glial cells. We addressed the role of astrocytic gap junctions in the development of astrocytic synchronization and propagation of seizures by application of the gap junction blocker carbenoxolone (CBX, 200 μM). Two different patterns of SLE activity was observed in the presence of CBX. In 41% of slices (7/17) CBX completely prevented epileptic activity (Figure 5A), indicating that inhibition of astrocytic synchronization through gap junctions may be a promising anti-epileptic strategy. In the other 10 of 17 slices CBX did not prevent SLE generation (Figure 5B), but still attenuated the incidence of seizures by significantly increasing the interictal interval after the first SLE from 368 ± 28 s in the absence of CBX to 523 ± 52 s in the presence of CBX (*p* = 0.006, *n* = 13 slices). The interictal interval after the second SLE further increased from 296 ± 32 s in the absence of CBX to 691 ± 80 s in the presence of CBX (*p* < 0.001, *n* = 13 slices) In accordance, CBX, applied after SLE1 inhibited the extent of astroglial synchronization as the average of maximal astroglial synchronization reached 59 ± 8%, 53 ± 5%, and 24 ± 5% in the first three consecutive SLEs, respectively (*n* = 13 slices). The synchronization in the third SLE is significantly different from the corresponding control SLE in the absence of drugs (44 ± 6%, *p* = 0.027).

Interestingly, we have observed that while CBX inhibited astrocytic synchronization during SLEs, it paradoxically increased the frequency of Ca^2+^ transients shortly after its administration in the first interictal period (Figures 5A,C) in both groups of SLE activity pattern. Although these Ca^2+^ events were not synchronized, they apparently contributed to seizure prevention, since both the frequency and average amplitude of these Ca^2+^ transients were linearly correlated to the length of the interictal period, hampering the appearance of SLEs even in the 10 of 17 slices in which further SLEs could be observed during CBX application (Figure 5D). Total elimination of SLEs in 7 of 17 slices was also coincided with high frequency (0.23 ± 0.07 1/min) and high peak amplitude (0.32 ± 0.11) of Ca^2+^ transients shortly after CBX application.

Although CBX is widely used to study the contribution of astrocytic gap junctions to different physiological processes, it blocks not only astrocytic, but also neuronal connexins (Molnár et al., 2011a; Verselis and Srinivas, 2013). Since inhibition of neuronal Cx36 may also result in reduced synchronization (Allen et al., 2011) and consequently in decreased epileptiform activity, it is desired to specifically inhibit Cx43, the major astrocytic connexins isoform. Unfortunately, genetic deletion of Cx43 leads to significantly altered neurogenesis and synaptic transmission (Lutz et al., 2009; Pannasch et al., 2011; Chen et al., 2012). Also, brain slices from Cx43 and Cx30 double knock-out mice have been shown to generate spontaneous epileptiform events possibly due to the chronic impairment of their Glu and K^+^ buffering capacity (Wallraff et al., 2006). Therefore, Cx43 KO mice are not suitable to investigate the acute effect of Cx43 inhibition. In the absence of specific inhibitor molecule, we opted to investigate the role of Cx43 gap junctional coupling by a specific antibody against its gating peptide segment (Sosinsky et al., 2007), which has previously been demonstrated to block Cx43 function (Molnár et al., 2011a). After the first SLE, slices were incubated with the Cx43 antibody in 1:100 dilution (7.5 μg/ml) for 10 min followed by washout. Similarly to the results obtained in the presence of CBX, specific Cx43 blockade by an antibody also led to complete elimination of seizures in 42% of slices (5/12; Figure 5E). Furthermore, in the other 7 of 12 slices, the interictal interval after the first SLE significantly increased from 289 ± 24 s under control conditions to 502 ± 124 s after Cx43 antibody application (*p* = 0.025, *n* = 9 slices). The interictal interval after the second SLE also significantly increased from 371 ± 30 s under control conditions to 582 ± 94 s after Cx43 antibody application (*p* = 0.012, *n* = 9 slices).

Therefore, inhibition of astrocytic synchronization by Cx43 inhibition clearly demonstrated to be anticonvulsive even after relatively short application time *in vitro*.

### Blockade of a Local Neuro-Glia Interaction

**Figure 6:**
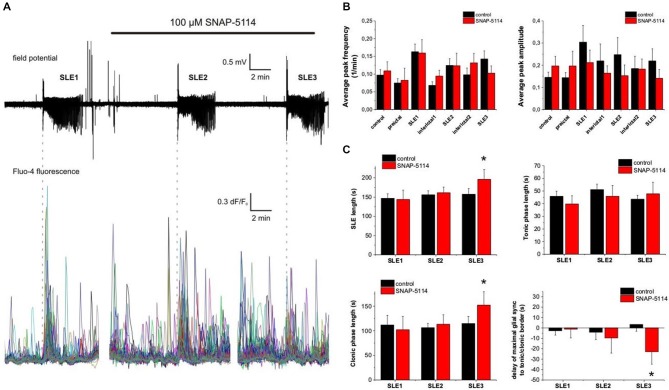
**Blockade of a local neuro-glia coupling process by SNAP-5114.** (**A**) (Top) Extracellular field potential recording of seizure-like events (SLEs) in the CA3 region in a hippocampal slice. (Bottom) Ca^2+^ transients measured by Fluo-4 fluorescence in CA3 astrocytes. Vertical dashed lines indicate the start of SLEs. (**B**) Average peak frequency (left) and peak amplitude (right) during the control period, the application of low-[Mg^2+^] ACSF (preictal) and the SLEs and interictal periods. (**C**) Average SLE length parameters: total length (top left), length of tonic (top right) and clonic (bottom left) phases. (Bottom right) Average delay of maximal glial synchronization time to the tonic/clonic transition under control conditions and in the presence of 100 μM SNAP-5114. Asterisks denote significant differences between the control conditions and SNAP-5114 application.

In addition to the long-range promotion of seizure-like activity, astrocytes are also able to locally modulate neuronal activity under epileptic conditions (Steinhäuser and Seifert, 2012; Crunelli et al., 2015; Héja, 2014). These local cross-talk processes also increase the likelyhood to initiate long-range Ca^2+^ waves, therefore promoting seizure propagation. One of the local neuro-glia cross-talk processes is the glial Glu/GABA exchange mechanism by which astrocytes convert neuronal excitation to tonic inhibition through changes in intracellular [Na^+^] (Héja et al., 2009, 2012). Blockade of this mechanism has been demonstrated to significantly enhance (by approximately 100%) the EAAT-mediated increase in the intracellular glial Na^+^ level (Héja et al., 2012) which can consequently trigger fast Ca^2+^ transients through the reverse action of the Na^+^/Ca^2+^ exchanger. We investigated whether the locally initiated Ca^2+^ elevation by Glu/GABA exchange blockade may trigger astrocytic synchronization. We used the specific inhibitor of glial GAT-2/3 transporters, SNAP-5114 (100 μM) to block the glial Glu/GABA exchange. SNAP-5114 was applied following the first SLE (Figure 6A). In the presence of SNAP-5114 neither the frequency nor the average amplitude of the Ca^2+^ transients changed significantly during SLEs and the interictal periods (Figure 6B). The average of maximal glial synchronization reached 57 ± 6%, 47 ± 8%, and 49 ± 6% in the first three consecutive SLEs, respectively (*n* = 10 slices) which was not significantly different from corresponding control SLEs in the absence of drugs.

In addition, the extent of astrocytic synchronization also did not change compared to control SLEs. However, we observed, that the correlation between the timing of maximal glial synchronization and the electrographic tonic/clonic transition detached, the maximal synchronization appeared significantly earlier than the tonic/clonic transition (Figure 6C). This uncorrelation was corresponded to a specific increase in the length of the clonic phase of the third SLE (Figure 6C).

These findings, therefore, suggest that local cross-talk between neurons and astrocytes do not necessarily evolve into long-range astrocytic synchronizations.

## Discussion

Astrocytes are increasingly recognized as active partners of the tripartite synapse and are important modulators of neuronal activity both in physiological and pathophysiological states. In particular, several pathways have been revealed in the recent years through which astrocytes expose either anti- or pro-epileptic effects (Steinhäuser and Seifert, 2012; Crunelli et al., 2015; Héja, 2014). Importantly, astrocytes have the capability to sense neuronal activity, distribute the signal to long ranges and to modulate neuronal activity at far location afterwards. Here we investigated whether astrocytic Ca^2+^ transients, an established readout of astrocytic activity follows the activation pattern of neurons monitored by electrophysiological or optical methods. The experiments in this paper demonstrate that astrocytes show highly synchronized activity after the onset of recurrent neuronal epileptiform discharges which evolves into astrocytic seizure-like events (SLEs). Importantly these astrocytic SLEs appeared to contribute to seizure propagation, since blockade of intercellular gap junctional communication between astrocytes decreased not only the astrocytic synchronization, but also inhibited or completely prevented the generation of SLEs.

### Glial Synchronization is Coupled to Neuronal Activity During Seizure-Like Events

Numerous pathways exist through which astrocytes and neurons bidirectionally influence each other’s activity, including the release of neuro- and gliotransmitters (Parpura et al., 1994) or modulation of the ionic concentrations in their mutual intermediate environment, the extracellular space (Kovács et al., 2000; Amzica and Massimini, 2002). The complex network formed by glial and neuronal cells, therefore, have the potential to display coherent, synchronized activities. Despite of this probability, spontaneous Ca^2+^ signals of astrocytes do not show any significant synchronization under control conditions. In contrast, we have shown that after the onset of SLE, a massive synchronized activity is emerged in both neurons and astrocytes. Noteworthy, we have detected astrocytic Ca^2+^ events in the soma and increase in the frequency of somatic Ca^2+^ events may just be a consequence of increased spreading of spontaneous Ca^2+^ fluctuations in astrocytic processes as suggested by Wu et al. (2014). Whether new Ca^2+^ events are triggered during SLEs or the spreading of existing ones are raised, the increased appearance of somatic Ca^2+^ transients delineates intensified astroglial activity that will increase the probability of intercellular activation in the astroglial network. This kind of simultaneous activation demonstrates that, in addition to the widely studied synchronization among neurons, astrocytes also display synchronous activity during epileptic seizures. Importantly, cross-correlation analysis showed that synchronization occurs not only among neurons or astroglial cells, but also between the two distinct cell populations, demonstrating the coupled dynamics of neuronal and astrocytic activity.

Since astrocytic synchronization did not appear either in normal ACSF or in low-[Mg^2+^] ACSF in the preictal phase, it is an important issue to resolve, what is the threshold of neural activity that triggers synchronized Ca^2+^ signaling in astrocytes. Previous studies debated whether astrocytic activation is associated with preictal (Carmignoto and Haydon, 2012), interictal (Tian et al., 2005) or ictal discharges (Gómez-Gonzalo et al., 2010). Occasionally we observed increased glial Ca^2+^ signaling corresponding to late preictal or interictal discharges, but these events never evolved to synchronized astrocytic Ca^2+^ oscillations, supporting the view (Gómez-Gonzalo et al., 2011) that long-range astrocytic Ca^2+^ signaling contributes predominantly to the ictal-like discharges. Besides, significant astrocytic synchronization never appeared before the seizure onset, indicating that astrocytic synchronization does not initiate SLEs, its role may be considered rather in the maintenance of recurrent epileptiform activity. We may infer therefore that astrocytic synchronization requires a robust preceding neuronal activity.

### Local Ca^2+^ Enhancements do not Necessarily Induce Astroglial Synchronization

Since enhanced, synchronized neuronal activity appears to be a prerequisite for the astrocytic synchronization, it is an important question of how the neuronal activity is transmitted to astrocytes and whether local cross-talk between neurons and astrocytes are able to trigger long-range astroglial synchronization. Neuronal activity can be detected by astrocytes by various routes, like direct activation of astrocytic glutamate (Glu) or GABA receptors, Na^+^ influx through astroglial Glu transporters or passive uptake of extracellular K^+^ originating from neurons. As specific inhibitors of astroglial neurotransmitter receptor subtypes are currently not available and inhibition of astroglial Glu or K^+^ uptake would lead to overexcitation of neurons, the contribution of these pathways to the development of astrocytic synchronization cannot be pharmacologically isolated without seriously disturbing neuronal activity. Therefore, we opted to intervene in the neuro-glia signaling by the blockade of a local, *in situ* cross-talk of neurons and astrocytes, the neuroprotective astrocytic Glu/GABA exchange mechanism (Héja et al., 2009, 2012; Dvorzhak et al., 2013; Unichenko et al., 2013). We have previously shown that inhibition of this process by blockade of the astroglial GABA transporter GAT-2/3 leads to increased intracellular Na^+^ concentration (Héja et al., 2012) that may evoke fast Ca^2+^ signals through the reverse action of the Na^+^/Ca^2+^ exchanger (Kirischuk and Kettenmann, 1997). Local initiation of Ca^2+^ elevation by this way, however, did not change either the frequency or the amplitude of global astrocytic Ca^2+^ transients, nor did it trigger astrocytic synchronization, suggesting that local elevation of astrocytic Ca^2+^ via Na^+^/Ca^2+^ exchange does not necessarily transform into long-range Ca^2+^ signals. The specific increase in the clonic phase length due to the blockade of the Glu/GABA exchange mechanism (Héja et al., 2012 and this work) however, resulted in faster appearance of maximal astrocytic synchronization. The correlation between the maximal astroglial synchronization and the transition from the tonic to clonic phase is in accordance with the maximal decrease of the extracellular Ca^2+^ level at the electrographic tonic/clonic transition (Kovács et al., 2000), suggesting that neuronal Ca^2+^ dynamics may be transmitted to astrocytes through the extracellular space.

### Gap Junction Mediated Long-Range Astroglial Synchronization Contributes to Seizure Propagation

Distance dependance of the synchronization between neurons and astrocytes suggests the possibility that local association of the two cell types may propagate through the glial syncytium and therefore supports the long range development of seizures. A major player in forming the astrocytic syncytium is the direct physical contact between astrocytes by gap junction proteins. Gap junctions are penetrable for several ions and small molecules, contributing to the distribution of metabolites and signaling agents. Distributions of nutrients is suggested to be pro-convulsive, as it provides supply for the maintenance of seizures (Gigout et al., 2006; Bostanci and Bağirici, 2007). In contrast, buffering of K^+^ and Glu is considered to be anticonvulsive (Wallraff et al., 2006) by reducing excess excitability. The dual role of gap junctions in epilepsy is generally addressed by a fast-onset anticonvulsive effect corresponding to K^+^ and Glu buffering and a delayed proconvulsive effect due to the maintenance of neuronal energy supply (Steinhäuser and Seifert, 2012).

To elucidate the apparently contradictory effects of gap junctions in SLE generation and maintenance, we investigated whether gap junction blockade by CBX directly affects astrocytic synchronization. As expected, CBX inhibited astroglial synchronization. Importantly, CBX also prevented SLE generation in 7 of 17 slices and increased the interictal interval in the remaining 10 slices, suggesting that the intercellular communication between astrocytes contributes to long-range propagation of synchronized activity. In addition to the non-specific gap junction blocker CBX, specific inhibition of the astrocytic Cx43 gap junction by an antibody against its gating peptide segment also resulted in complete elimination of SLEs or an increase of the interictal interval, excluding the possibility that CBX inhibited SLEs by acting on the neuronal connexins. Blockade of astrocytic gap junctions is able to interfere with epileptic activity due to reduction of glial synchronization or inhibition of nutrient distribution. Distribution of glucose in the astrocytic syncytium, however, is a slow process that requires 30–60 min to transport glucose in a 70 × 70 μm area (Rouach et al., 2008). Besides, it was demonstrated that the Cx43 mimetic peptide Gap 27 requires a very long incubation time (>10 h) to significantly reduce the gap junction-mediated distribution of a fluorescent glucose derivative and to consequently affect the characteristics of spontaneous SLEs (Samoilova et al., 2008), despite the fact that the same peptide has been shown to effectively reduce the calcium wave size by 60% after a much shorter application (30 min; Braet et al., 2003). Since CBX and the Cx43 antibody exerted their anticonvulsive effect in a relatively short time, we propose that their effects are mediated by the inhibition of synchronized Ca^2+^ transients. Interestingly, CBX also significantly increased the frequency of unsynchronized Ca^2+^ transients in the interictal phase. These signals may correspond to the inability of astrocytes to distribute K^+^, which results Ca^2+^ release from internal stores in single astrocytes in an uncorrelated manner. Indeed, we showed that the frequency of these Ca^2+^ signals positively correlated with the length of interictal periods, suggesting that local, unsynchronized Ca^2+^ transients inhibit, while long-range, synchronized Ca^2+^ transients contribute to seizure propagation. Gap junction function, therefore, can be proconvulsive both on the long and short term by the distribution of energy metabolites (Rouach et al., 2008; Samoilova et al., 2008) and by the potential promotion of astrocytic synchronization (this work).

In conclusion, we have shown that a significant synchronization of astrocytes emerges during ictal-like discharges coupled to neuronal synchronization. The astrocytic synchronization mediated by gap junctions contributes to the propagation of seizures, providing an explanation why CBX treatment can be beneficial for *in vivo* epilepsy conditions (Bostanci and Bağirici, 2007).

## Conflict of Interest Statement

The authors declare that the research was conducted in the absence of any commercial or financial relationships that could be construed as a potential conflict of interest.
